# Limitation of Cytokinin Export to the Shoots by Nucleoside Transporter ENT3 and Its Linkage with Root Elongation in Arabidopsis

**DOI:** 10.3390/cells10020350

**Published:** 2021-02-08

**Authors:** Alla Korobova, Bulat Kuluev, Torsten Möhlmann, Dmitriy Veselov, Guzel Kudoyarova

**Affiliations:** 1 Laboratory of Plant Physiology, Ufa Institute of Biology, Ufa Federal Research Centre, RAS, 450054 Ufa, Russia; muksin@mail.ru (A.K.); veselov@anrb.ru (D.V.); 2 Institute of Biochemistry and Genetics, Ufa Federal Research Centre, RAS, 450054 Ufa, Russia; kuluev@bk.ru; 3 Biological Department, Bashkir State University, 450076 Ufa, Russia; 4 Department of Biology, University of Kaiserslautern, 67663 Kaiserslautern, Germany; moehlmann@biologie.uni-kl.de

**Keywords:** *Arabidopsis thaliana*, ENT3, exogenous cytokinin riboside, endogenous cytokinins, membrane transporters, root elongation

## Abstract

The trans-membrane carrier AtENT3 is known to transport externally supplied cytokinin ribosides and thus promote uptake by cells. However, its role in distributing either exogenous or endogenous cytokinins within the intact plant has not hitherto been reported. To test this, we used *ent3-1* mutant Arabidopsis seedlings in which the gene is not expressed due to a T-DNA insertion, and examined the effect on the concentration and distribution of either endogenous cytokinins or exogenous trans-zeatin riboside applied to the roots. In the mutant, accumulation of endogenous cytokinins in the roots was reduced and capacity to deliver externally supplied trans-zeatin riboside to the shoots was increased suggesting involvement of equilibrative nucleoside (ENT) transporter in the control of cytokinin distribution in the plants. Roots of *ent3-1* were longer in the mutant in association with their lower cytokinin concentration. We concluded that the ENT3 transporter participates in partitioning endogenous cytokinins between the apoplast and the symplast by facilitating their uptake by root cells thereby limiting cytokinin export to the shoots through the xylem. Dilution of the mineral nutrient solution lowered endogenous cytokinin concentration in the roots of both wild type (WT) and *ent3-1* plants accompanied by promotion of root elongation. Nevertheless, cytokinin content was lower, while roots were longer in the *ent3-1* mutant than in the WT under either normal or deficient mineral nutrition suggesting a significant role of ENT3 transporter in the control of cytokinin level in the roots and the rate of their elongation.

## 1. Introduction

Regulation of cytokinin concentration and distribution in plants is important for the control of their adaptation to environmental changes. A decline in the content of these hormones in shoots has been frequently detected under water deficiency in tobacco [1] and tomato [2], as well as under deficiency of mineral nutrients in Arabidopsis [3], wheat [4], barley [5], and rice [6]. Cytokinins are known to exert opposite effects on shoots and roots, promoting the shoot growth, while inhibiting the root growth [7]. Consequently, a reduction in cytokinin levels detected under deficiency of mineral nutrients or water [8] should inhibit shoot growth, while at the same time promoting root growth. Cytokinins have been shown to inhibit root elongation [9] and accumulation of root biomass [10] and consequently a decline in root cytokinin concentration is expected to activate root growth [6]. There are various mechanisms regulating cytokinin concentration under the changing environment. Thus, activation of cytokinin oxidases, resulting in inactivation of these hormones, served as a cause of the decline in cytokinin content in wheat plants exposed to diluted nutrient solution [4]. Addition of nitrates to the nutrient medium increased cytokinin level in plants brought about by up-regulation of *IPT* genes responsible for the synthesis of cytokinins in roots of maize [11] and leaves of Arabidopsis [12].

An important role for the regulation of cytokinins levels in plants is attributed to their transport from roots to shoots [13]. Although expression of *IPT* genes was detected not only in the roots but also in the shoots [12], the decline in capacity of roots for cytokinin export resulted in reduced accumulation of cytokinins in the shoots [14], confirming the importance of roots for the supply of shoots with these hormones. In these experiments, disturbance of cytokinin distribution between roots and shoots was due to mutation of the gene coding for ABCG14, an ABC-transporter enabling the export/efflux of these hormones from root cells, which contributes to cytokinin loading into the xylem. The shootward transport of root-derived cytokinins was significantly suppressed in rice plants with mutation in ABC transporter OsABCG18 [15].

Alongside ABC carriers, transport of cytokinins across membranes may be performed by purine permeases (PUPs) specialized in transport of nitrogenous bases [16] and equilibrative nucleoside (ENT)-transporters capable of transferring their ribosides [17]. Unlike ABC proteins, PUPs and ENT transporters are believed to support cytokinin uptake by the cells and not outflow of the hormones from the cells [18]. Recently attention has been drawn to the role of PUP-carriers for the transport of cytokinins [19,20], but there is far less information concerning transporters of cytokinin ribosides. Although free cytokinin bases are known to be the active form of cytokinins, ribosylated cytokinins are the dominant form of these hormones present in the xylem sap and transported from roots to shoots [21]. Therefore, ENT transporters specialized in the trans-membrane transfer of ribosylated nitrogenous bases are likely to be involved in the transport of cytokinins from roots to shoots. However this possibility has not been studied so far. It is of interest that corresponding reporter constructs co-localized expression of the genes coding for IPT [12], ENT [22], and nucleoside hydrolase, NSH1 [23] in the root vasculature. The results suggest coordinated control of cytokinins synthesis, metabolism, and transport brought about by co-localization of corresponding participants of this network.

In the present work we have focused on ENT3, as this carrier represents the main nucleoside importer at the plasma-membrane in Arabidopsis. Studies of the uptake of radiolabeled nucleosides into yeast (*Saccharomyces cerevisiae*) cells and electrophysiological measurements on Xenopus oocytes expressing ENT3 clearly showed involvement of ENT3 in high affinity nucleoside/proton symport [24,25]. In addition, reduced uptake of nucleosides by hypocotyl explants of an ENT3 T-DNA insertion mutant (*ent3-1*) was observed in seedlings, leaf discs, and roots from hydroponically grown plants [25]. Most importantly, the efficiency of uptake of the cytokinin isopentenyladenine riboside (iPR) by hypocotyls was reduced more than 40% in *ent3-1* [26]. However, information about physiological role of this transporter in terms of cytokinin function is limited to the data showing decreased sensitivity of root growth of the *ent3-1* mutant to exogenous cytokinin ribosides [26]. Since ENT3 is highly expressed in the root vasculature of seedlings carrying the *AtENT3*-promoter–GUS construct [22], this carrier is likely to function in roots and to influence cytokinin export to the shoots. However, until now no indication of a physiological role of any ENT in the control of concentration of endogenous cytokinins in the roots and their distribution between roots and shoot has been provided.

The aim of the present study was to unravel a possible role of ENT3 in the uptake of cytokinins into root cells and its linkage with corresponding alterations in cytokinin distribution between roots and shoots and effects on root growth. To do so, the content and distribution of endogenous and exogenous cytokinins were quantified in the *ent3-1* mutant and its wild type, Col. In addition, expression of cytokinin biosynthetic genes encoding isopentenyl transferase (IPT), activity of cytokinin oxidases, and root growth of *ent3-1* mutant and its wild type, Col were studied. To unravel possible involvement of ENT in plant responses to availability of mineral nutrients, plants were either supplied with sufficient mineral nutrients or grown under nutrient limitation. We described for the first time the physiological role of ENT3 transporter in the regulation of cytokinin level (both the uptake and distribution) in shoots and roots and its effects on root elongation in Arabidopsis. The results suggest that ENT3 transporter distributes endogenous cytokinins between the apoplast and the symplast by facilitating cytokinin uptake by root cells, thereby preventing their long distance transport from roots to shoots.

## 2. Materials and Methods

### 2.1. Plant Material and Growth Conditions

The experiments were performed with *Arabidopsis* plants (*Arabidopsis thaliana* [L.] Heynh.) of wild type (WT) ecotype Columbia (Col) and *ent3-1* (SALK_131585) mutant impaired in the function of ENT3 (AGI code; At4g05120) nucleoside transporter [22]. After stratification on wet filter paper in Petri dishes for 3 days at 4 °C, the seeds were transferred to 100 mL vessels filled with sand saturated with 0.1-strength Hoagland–Arnon solution and grown in an MLR-350H controlled-climate chamber (Sanyo, Osaka, Japan) for 28 days at a temperature of 23/19 °C (day/night), relative air humidity of 80%, photosynthetically active radiation of 120 μmol/(m^2^ s) PAR, and 16-h-long photoperiod as described in [27]. A 0.1-strength of Hoagland–Arnon solution was chosen in preliminary experiments as optimal for maintaining Arabidopsis growth. Every day, the plants were supplied with 3 mL of Hoagland–Arnon solution and distilled water by the weight of vessels for the maintenance of sand hydration at 60% of full water capacity as described earlier [28]. Effects of the shortage of mineral nutrients on cytokinin content and root elongation was studied in experiments, where sand substrate was washed with 75 mL of 0.01-strength Hoagland–Arnon solution taken in a volume five times greater than that of the solution in containers used for plant growing (4-week-old plants). To study the uptake of exogenous cytokinins by the plants, 3-week-old seedlings were placed into container with microplates with holes made in their bottom. Microplates were floating over the surface of aerated nutrient solution in 5-L-containers. One week after placing the plants into hydroponics, trans-zeatin riboside was added to the solution to yield final concentration of 4 × 10^−7^ M.

Hormone sampling for cytokinins assay was performed 1 and 5 days after addition of zeatin riboside and sand washing, correspondingly. Activity of cytokinin oxidases and levels of the transcripts of isopentenyl transferase genes were measured in 4-week-old plants grown in the sand. Root length was estimated 7 days after sand washing.

### 2.2. Cytokinin Extraction, Purification, and Immunoassay

Shoots and roots of Arabidopsis plants were homogenized in 80% ethanol and kept overnight at 4 °C. After filtration, all traces of ethanol were removed by vacuum evaporation and cytokinins from aqueous residue were purified as described [29,30]. In short, the hormones were concentrated on C18 column (Waters, Milford, MA, USA) and eluted with 5 mL of 80% ethanol. The dry residues dissolved in small volumes of ethanol were applied to TLC plates and were developed in a mixture of 2-butanol and NH_4_OH for separation of cytokinin bases and their derivatives. This procedure enabled separation and assay of cytokinin nucleotide (Rf 0–0.1), cytokinin glucosides (Rf 0.1–0.2), and riboside of zeatin (ZR, Rf 0.4–0.5). Anti-*trans*-ZR rabbit serum was used for the enzyme-linked immunosorbent assay of cytokinins and its specificity has been described previously [2,31,32]. In short, enzyme immunoassay was performed with the protocol in which a conjugate of ZR to ovalbumin is absorbed onto the solid phase. A mixture of standard or sample plus specific serum was added to each well and incubated. Unbound rabbit antibodies were washed away and goat anti-rabbit IgG, conjugated to peroxidase, was incubated with the adsorbed antigen-antibody complex. All wells were again washed and the substrate solution consisting of o-phenylene-diamine was added. Color developed was quantitated at 492 nm with a microphotometer. Antibodies had high immunoreactivity towards corresponding zeatin and their derivatives (ribosides, 9-N-glucosides, and nucleotides) and low cross-reactivity to cis-Z and iP-type cytokinins (less than 1%). This method has proven to be reliable by testing its results against physico-chemical assay [28,29,33].

### 2.3. Measurement of Cytokinin Oxidase Activity

Activity of cytokinin oxidase was detected as described previously [4,34]. Leaves were homogenized in 0.1 M imidazole buffer (pH 7.1) and centrifuged at 12,000× *g* for 30 min. To separate hormones from high molecular weight fraction, equal volume of saturated solution of ammonium sulfate was added to the supernatant and the pellet was resuspended in 0.1 M imidazole buffer (pH 7.1). Then, 30 ng of cytokinin isopentenyl adenine (iP) added as a substrate to the suspension followed by incubation for 3 h at 37 °C. Cold ethanol was added to each sample to yield final 60% concentration (*v*/*v*), the resulting suspension was kept at 4 °C for 40 min and then centrifuged at 5000× *g* for 10 min. The decline in iP content in supernatant was detected with the help of immunoassay using antibodies against iPA. The absence of iP conversion to zeatin or dihydrozeatin was checked with immunoassay using corresponding antibodies. The presence of imidazole and Cu^2+^ accelerated the decay of iP confirming that the decline in iP level was due to its conversion to non-immunoreactive adenine catalyzed by cytokinin oxidase.

### 2.4. Expression Analysis

PCR with real-time product detection was performed as described previously [35]. RNA was isolated from the roots and leaves of plants of original ecotype and *ent3-1* mutant with Trizol. Total RNA was treated with RQ1 DNAase free of RNAases (Promega; http://www.promega.com/ (accessed on 18 December 2020)), then reverse transcription was performed using oligo (dT) primer and M-MuLV-reverse transcriptase (NEB New England Biolabs, Ipswich, MA, USA). In order to quantify transcripts of the genes encoding isopentenyl transferase (*AtIPT1*, *AtIPT3*, *AtIPT5*, and *AtIPT7*), we used primers described earlier [12]. Realtime RT-PCR was conducted in a Rotor-GeneTM 6000 thermocycler (Corbett Research, Mortlake, NSW, Australia) in the presence of intercalator SYBR Green. As an internal standard, we used a household gene of Arabidopsis *AtACT2* (At3g18780). Calculations were made using the 2(-Delta C(T)) method [36], and the mRNA level of the elongation factor *AtACT* was defined as 100%.

### 2.5. Statistics

The experiments were repeated three times with 3–20 replicates. Data were expressed as means ± SE, which were calculated in all treatments using MS Excel. A significant (at *p* < 0.05) difference between the means was calculated using *t*-test

## 3. Results

### 3.1. Concentration of Cytokinins and Their Metabolism (Cytokinin Oxidase Activity and Expression of AtIPT Genes) in ent3-1 Mutant and Col Plants

**Figure 1:**
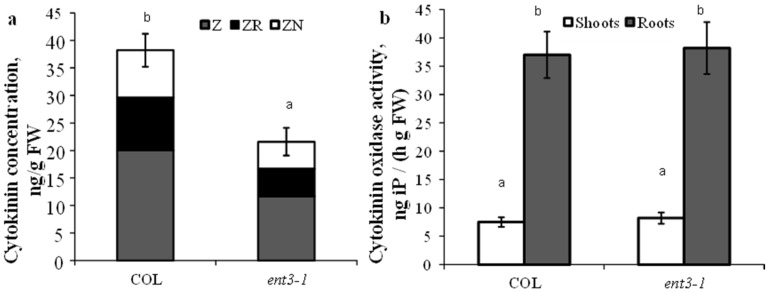
Concentration of zeatin (Z), zeatin riboside (ZR), and zeatin nucleotide (ZN) in roots (**a**) and cytokinin oxidase activity (**b**) in hydroponically grown 4-week-old *ent3-1* Arabidopsis mutant and its parent ecotype Columbia (Col). Statistically differing means (n = 6) are indicated with different letters (*p* < 0.05, Student’s *t*-test).

Content of cytokinins in the roots of hydroponically grown mutant plants (*ent3-1*) was lower than in WT (Col) plants (Figure 1a). This regularity has also been detected in plants grown in the sand. Activity of cytokinin oxidase either in shoots or roots was the same in the mutant and WT plants and consequently the decreased level of cytokinins detected in the roots of *ent3-1* plants could not be due to the activity of cytokinin degradation in the plants (Figure 1b).

**Figure 2:**
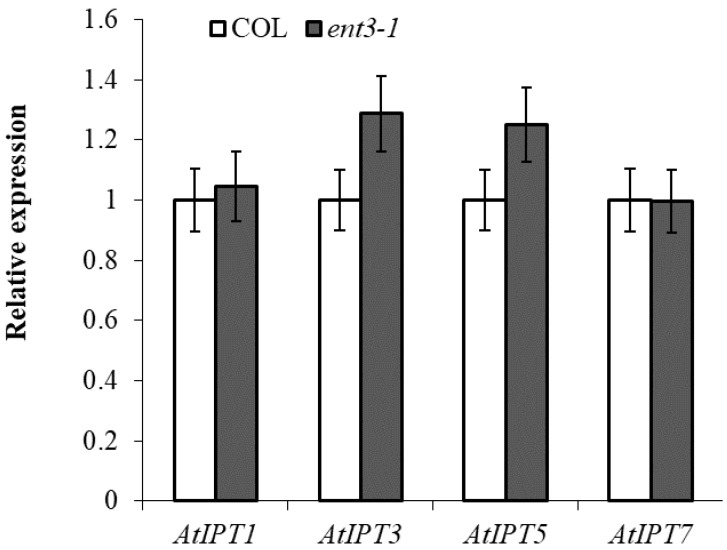
Level of transcripts of *IPT1* (AT1G68460.1)*, IPT3* (AT3G63110.1)*, IPT5* (AT5G19040.1), and *IPT7* (AT3G23630.1) in the roots of 4-week-old *ent3-1* mutant and its parent ecotype Columbia (Col). Average expression levels from three replicates were normalized to actin with wild type scaled to 1 (n = 3).

RT-PCR analysis of the transcripts of *IPT*-genes known to be expressed in the roots [12] showed that expression of the genes was not lower in *ent3-1* than in Col thereby excluding possibility of attributing the decreased level of cytokinins in the roots of the mutant to their reduced synthesis (Figure 2).

### 3.2. Concentration of Cytokinins in Plants Treated with Zeatin Riboside

**Figure 3:**
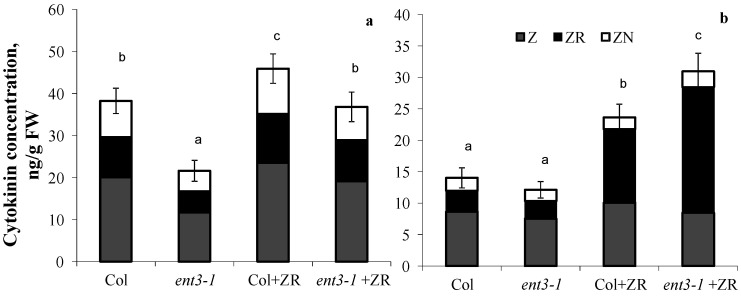
Concentration of zeatin (Z), zeatin riboside (ZR), and zeatin nucleotide (ZN) in roots (**a**) and shoots (**b**) of hydroponically grown 4-week-old *ent3-1* Arabidopsis mutant and its parent ecotype Columbia (Col) 1 day after zeatin riboside application (Col + ZR, *ent3-1*+ZR) to 0.1 Hoagland–Arnon nutrient solution (to yield 4 × 10^−7^ M). Statistically differing means (n = 6) are indicated with different letters (*p* < 0.05, Students *t*-test).

In plants grown hydroponically in the solution without zeatin riboside, root concentration of cytokinins was higher in WT (Col) than in the mutant (*ent3-1*) (Figure 3a). Although addition of zeatin riboside to the nutrient solution increased concentration of cytokinins in plants of both genotypes, concentration of these hormones was still higher in the roots of Col than *ent3-1.* The opposite pattern was detected in the shoots (Figure 3b)—addition of zeatin riboside resulted in greater increase in cytokinin concentration in shoots of the mutant than in WT. Furthermore, plant treatment with zeatin riboside most significantly influenced the content of zeatin riboside in shoots, whose percentage out of the total content of measured cytokinins increased from 23% in the control of both genotypes (plants untreated with exogenous cytokinins) to 50% in cytokinin-treated Col and to 65%-in *ent3-1*.

### 3.3. Effects of Dilution of the Nutrient Solution on Cytokinin Concentration

**Figure 4:**
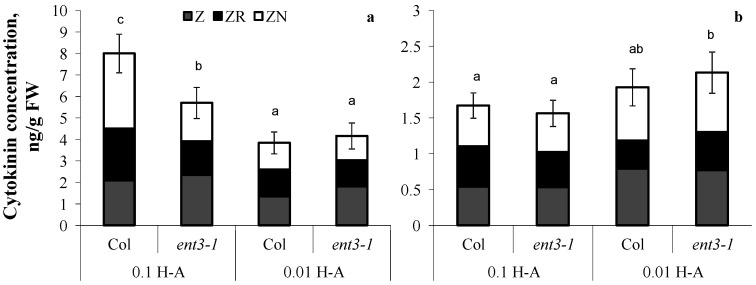
Concentration of zeatin (Z), zeatin riboside (ZR), and zeatin nucleotide (ZN) in roots (**a**) and shoots (**b**) of 5-week-old *ent3-1* Arabidopsis mutant and its parent ecotype Columbia (Col) grown in vessels filled with sand saturated with 0.1-strength Hoagland–Arnon solution and sampled 5 days after dilution (0.01 H-A) of optimal 0.1 strength Hoagland–Arnon nutrient solution (0.1 H-A). Statistically differing means (n = 6) are indicated with different letters (*p* < 0.05, Students *t*-test).

Dilution of the nutrient solution resulted in a decline in cytokinin content in the roots of both genotypes (Figure 4a) grown in sand. The percentage of zeatin riboside decreased from 30 to 20% out of the total sum of measured cytokinins in the plants of both genotypes.

**Figure 5:**
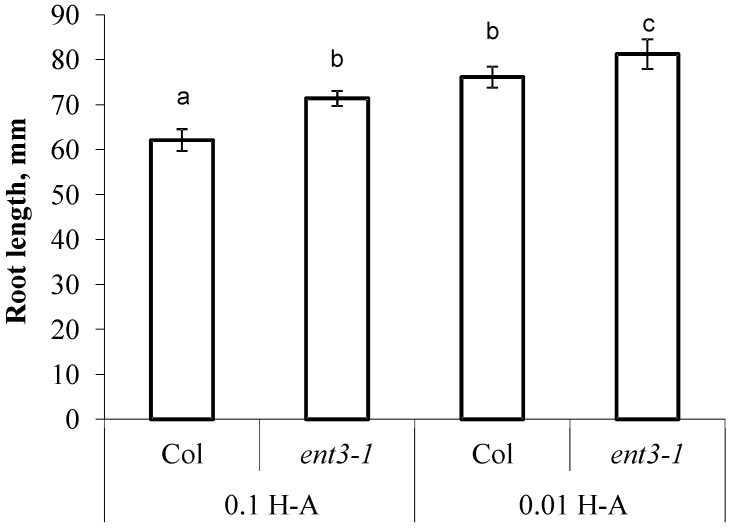
The length of the primary root of 5 weeks old *ent3-1* Arabidopsis mutant and its parent ecotype Columbia (Col) 7 day after dilution (0.01 H-A) of optimal 0.1 strength Hoagland–Arnon nutrient solution (0.1 H-A). Statistically differing means (n = 20) are indicated with different letters (*p* < 0.05, Students *t*-test).

The decline in concentration of mineral nutrients was accompanied by activation of root elongation—in Col the roots were 22% longer than in the control (Col plants supplied with sufficient mineral nutrition) and in *ent3-1* roots were 14% longer than in the corresponding control (Figure 5). Col roots were shorter than those of *ent3-1* under both levels of mineral nutrition (Figure 5).

## 4. Discussion

ENT was characterized as a cellular importer of nucleosides including cytokinin ribosides [25,26]. Involvement of ENT in cytokinin transport was demonstrated in experiments with yeast cells expressing rice *OsENT2* [37]. Meanwhile, information about its involvement in the control of cytokinin distribution in plants as well as in the implementation of the growth-regulating function of cytokinins is still scarce. Before our experiments were performed, this information was limited to the data showing decreased sensitivity of the root growth to exogenous cytokinin ribosides in the *ent3-1* mutant [26]. The in vivo ENT3 function was supported by the study of double knockout plants in which both *ENT3* and the extracellular nucleoside hydrolase *NSH3* genes (*ENT3::NSH3*) were inactivated—these plants accumulated adenosine and uridine in the apoplast of leaves [38]. As *ENT3* is highly expressed in the root vasculature of seedlings carrying AtENT3-promoter–GUS construct, a similar function in roots is highly likely [22]. Roots are a site for the synthesis of cytokinin ribosides which are transport form of cytokinins and precursors of active cytokinin bases [39]). Accordingly, ENT3 might function in cellular uptake of cytokinin ribosides, their conversion or signaling purposes in roots, thereby prohibiting export to the shoot via the xylem. This assumption is supported by the present experiments. A need for such uptake systems was proposed before [40] and a responsiveness of ENTs (ENT3 and ENT6) towards cytokinin treatment was shown [40]. However, until now no indication of a physiological role of any ENT in cytokinin metabolism was given. Reduced cytokinin riboside levels in roots of *ent3-1* mutants without or after ZR treatment shown here, fits nicely to a proposed function of ENT3 as cellular ZR importer in roots (Figure 3). Altered cytokinin synthesis or degradation as a reason for the observation of reduced cytokinin riboside levels in roots of the *ent3-1* mutant were ruled out as corresponding activities of CKX and expression of IPT genes were unchanged by the mutation.

Experiments with the uptake of exogenous zeatin riboside revealed that the decreased capacity of the *ent3-1* mutant for accumulation of cytokinins in roots contributes to increased export of these hormones to the shoots. This was suggested by the lower level of overall accumulation of cytokinins in the roots and the increased level of these hormones in shoots of the *ent3-1* mutant treated with exogenous zeatin riboside (as compared to Col). Another evidence of higher delivery of zeatin riboside to the shoots of the mutant is in greater increase in the percentage of this form of cytokinins in the shoots of the mutant treated with exogenous zeatin riboside. It was increased up to 65% out of total measured cytokinin content in the *ent3-1* mutant, while zeatin riboside reached only a 50% level in Col. Sun et al. [26] detected reduced uptake of iPR and ZR by the mutant. Nevertheless, in those experiments uptake of ribosides by the mutant was measured in hypocotyls and changes in distribution of cytokinins between roots and shoots have been observed for the first time in the present work.

It has been shown by us previously that the outflow of exogenous zeatin to the shoots of wheat plants is increased as a result of protonophore-dependent inhibition of secondary active uptake of cytokinins by root cells resulting in higher cytokinin concentration in the apoplast of the xylem vessels [29]. Present experiments suggest that uptake of cytokinins by root cells enables retention of cytokinins in the root cells of the vasculature, where ENT3 has been shown to be located in the previous experiments [26]. This process is likely to prevent cytokinin export to shoots through the xylem. Although in the previous experiments we were likely to inhibit PUP transporters, Arabidopsis ENT works in a similar way, i.e., as riboside-proton cotransporters. In accordance, we can interpret the present results as suggesting that knocking out the gene coding for the transporter of ribosides decreases capacity of root cells to retain cytokinins in the root cells contributing to their apoplast loading into xylem and transport to the shoots through xylem. The choice of trans-zeatin for supplying roots with exogenous cytokinin in our experiments is supported by information that trans-zeatin-type species are the major form in xylem sap [39], while we used antibodies raised against trans-zeatin riboside having low cross-reactivity to cis-zeatin forms.

**Figure 6:**
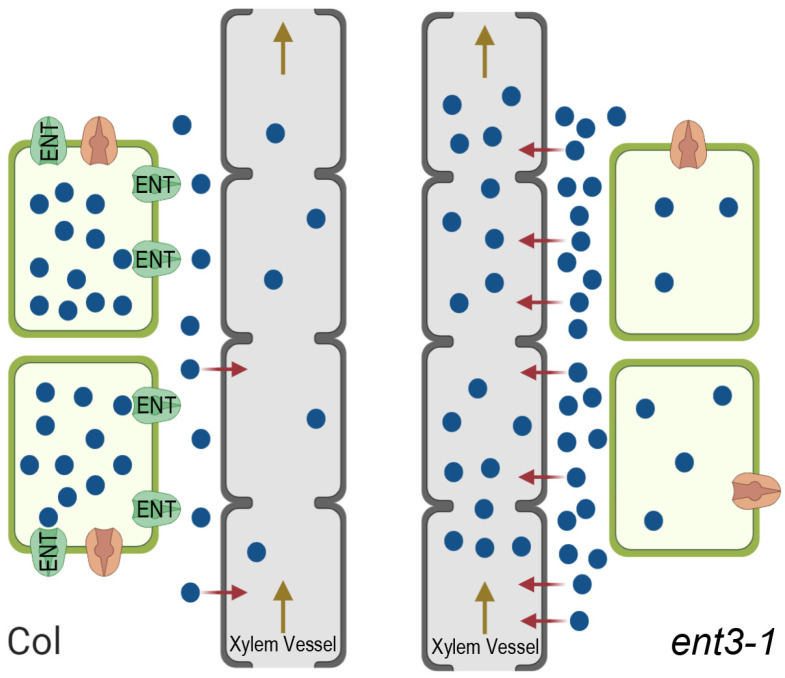
Scheme showing ENT-mediated uptake of externally supplied cytokinin riboside (blue circles) by living root cells, their passive loading into the dead xylem vessels (red arrows), and their flow to the shoot within the transpiration stream (brown arrow) in wild type Col plants (left) and *ent3-1* mutant (right). In Col, uptake and retention of cytokinin ribosides by living root cells occurs at a high rate resulting in a decreased apoplastic concentration, low loading into xylem vessels through the apoplast route, and reduced export to the shoots with the transpiration stream. In the *ent3-1* mutant cellular uptake of cytokinin riboside is reduced resulting in increased apoplast concentration, higher loading into the xylem apoplast, and greater export to shoots. Equivalent effects on endogenous cytokinins would indicate that the fully functional ENT3 carrier helps suppress losses to the shoot thereby maintaining the cytokinin titer of growing root cells at optimal levels.

This hypothesized mechanism is illustrated in Figure 6. The importance of the apoplast pathway with a transpiration stream for the delivery of zeatin riboside to the shoots has been demonstrated in experiments with the uptake of this hormone by wheat plants showing its decline in the plants under decreased transpiration, i.e., under conditions limiting apoplast pathways [41]. The effect of ENT on cytokinin transport to the shoots suggested by the present experiments is likely to be opposite to that of ABCG14 transporters. While the latter contributes to xylem loading of cytokinins and thereby enables their transport to shoots and the decline in cytokinins accumulation in roots [14], ENT3, on the contrary, is likely to decrease cytokinins delivery to shoots due to the ENT-driven uptake of these hormones by root cells bringing about the retention of cytokinins in roots.

The response to the deficit in mineral nutrients was similar in the plants of both genotypes. We detected a decline in root cytokinins of either *ent3-1* or Col accompanied by acceleration of root elongation. Similarity in the responses of the mutant and WT plants to dilution of the nutrient solution suggests that the membrane transport of cytokinin ribosides does not play a significant role in the decline in root cytokinins level under the shortage of mineral nutrients. An indirect evidence of this is in a reduced percentage of zeatin riboside registered by us in the shoots of both genotypes (from 30 to 20% out of the sum of all measured cytokinins). Our results are in accordance with those of Sun and co-authors [26], who detected an increased sensitivity to exogenous cytokinin riboside under nitrogen deficit that may serve as indirect evidence of decreased level of this form of cytokinins in nitrate-deficient plants.

Roots of the *ent3-1* mutant were longer than those of Col, while nutrient deficiency activated their elongation. This response is in accordance with the initial level of cytokinins that was lower in the mutant than in WT as well as with the decline in the hormone level detected in the nutrient-deficient plants. This correspondence is explained by the inhibitory effect of cytokinins on root elongation [9] (longer roots were characteristic of the plants with decreased level of cytokinins [7]). The results are in accordance with those of Sun et al., who detected inhibition of root growth in the plants treated with cytokinin riboside [26] and decreased inhibitory effect of exogenous cytokinins on the *ent3-1* mutant. The latter regularity may be due to decreased capacity of the *ent3-1* mutant to accumulate cytokinins in roots revealed in our experiments bringing about the reduced inhibitory effect of cytokinin ribosides on the root growth.

Biochemical peculiarities have been previously studied in nitrate-deficient *ent3-1* plants [22]. As far as we know, growth responses of the mutant to the deficiency in mineral nutrients had not been studied before our experiments were performed. Although the growth response was found to be similar in the plants of both genotypes, longer roots of the *ent3-1* mutant than of WT plants detected either under normal mineral nutrition or its deficit suggest a significant role of the ENT3 transporter in the control of cytokinin levels in the roots and the rate of their elongation.

## 5. Conclusions

Thus, comparison of cytokinin concentration and distribution between shoots and roots of WT and plants with the mutated gene coding for ENT3 showed implication of this transporter in the regulation of cytokinin levels in the plants, thereby enabling cytokinin-dependent regulation of root elongation in *Arabidopsis*. The changes in cytokinin distribution between roots and shoots resulting from mutation of the *ENT3* transporter gene have not been detected previously and were observed for the first time in the present work. The results suggest that uptake of cytokinins by root cells facilitated by ENT prevents their long-distance transport from roots to shoots, while a decline in the functioning of the transporter contributes to cytokinins export to the shoots. Control of cytokinins accumulation in the roots brought about by ENT transporters is likely to be involved in the regulation of root growth. ENT3 may also participate in modulating the balance of CK distribution between shoots and roots, which is important for the coordination of development of both parts of plants.
